# Adequate Sample Sizes for a Three-Level Growth Model

**DOI:** 10.3389/fpsyg.2021.685496

**Published:** 2021-07-01

**Authors:** Eunsoo Lee, Sehee Hong

**Affiliations:** Department of Education, Korea University, Seoul, South Korea

**Keywords:** three-level growth model, sample size, intraclass correlation, Monte Carlo simulation study, multilevel (hierarchical) modeling

## Abstract

Multilevel models have been developed for addressing data that come from a hierarchical structure. In particular, due to the increase of longitudinal studies, a three-level growth model is frequently used to measure the change of individuals who are nested in groups. In multilevel modeling, sufficient sample sizes are needed to obtain unbiased estimates and enough power to detect individual or group effects. However, there are few sample size guidelines for three-level growth models. Therefore, it is important that researchers recognize the possibility of unreliable results when sample sizes are small. The purpose of this study is to find adequate sample sizes for a three-level growth model under realistic conditions. A Monte Carlo simulation was performed under 12 conditions: (1) level-2 sample size (10, 30), (2) level-3 sample size (30, 50, 100) (3) intraclass correlation at level-3 (0.05, 0.15). The study examined the following outcomes: convergence rate, relative parameter bias, mean square error (MSE), 95% coverage rate and power. The results indicate that estimates of the regression coefficients are unbiased, but the variance component tends to be inaccurate with small sample sizes.

## Introduction

In education, counseling and social science research, observations with a hierarchical structure are common (Raudenbush and Bryk, 2002). Students are nested within classrooms or schools, workers are nested within firms, and patients are nested within counselors. When data are sampled in a multi-stage manner or observations (i.e., students) are nested within groups (i.e., schools), modeling data by ignoring the clustering can lead to false inferences about the relations among variables in the model. Therefore, methods have been developed for addressing data that come from a hierarchical structure. One such method is referred to as multilevel modeling (MLM), hierarchical linear modeling (HLM), mixed models or random coefficients modeling (Raudenbush and Bryk, 2002). Multilevel models can be conceptualized as regression models at two levels; that is, units of observation at one level are nested in groups at a higher level. However, there are many situations in which not only are the observations are nested within groups, but also repeated measures are nested within groups (Curran et al., 2012). In previous studies, researchers were interested in examining the change of individuals within groups by using the three-level growth model. For example, McCoach et al. (2006) examined the effects of school factors on the growth in kindergarten students' reading achievement with a three-level growth model. Lutz et al. (2007) used a three-level growth model to assess the amount of variance in across-session change in the symptom intensity scores of patients who were seeing different therapists. In their study, 1,198 patients were treated by 60 therapists, with a median number of sessions of 61.

The three-level growth model is conceptually similar to a linear regression model in that an outcome variable is predicted from multiple covariates. However, to handle clustered data, the within-group or individual variance is partitioned into the component at the lower level and the between-group variance at the upper level. For example, level-1 is each student's growth in academic achievement over time, level-2 is the variation across students in academic achievements within a group, and level-3 is the variation in initial status and growth rate among schools.

The equation for level-1 model is as follows

(1) $$\begin{array}{r} Y_{tij}=\pi_{0ij}+\pi_{1ij}time_{tij}+e_{tij} \end{array}$$

where *Y*_*tij*_ is the academic achievement at time *t* for individual *i* in group *j*; π_0*ij*_ is the initial status for student *i* in school *j*, that is, the expected outcome for student *i* in school *j* when time point = 0; π_1*ij*_ is the growth rate for student *i* in school *j*; *time*_*tij*_ is the student level time predictor at time *t* for student *i* in school *j*; and *e*_*tij*_ is the residual associated with a student's score at a specific time point, which is assumed to be normally distributed with a mean of 0 and variance of $\sigma_{e}^{2}$. The level-2 equations are

(2) $$\begin{array}{r} \pi_{0ij}=\beta_{00j}+\gamma_{0ij} \end{array}$$

(3) $$\begin{array}{r} \pi_{1ij}=\beta_{10j}+\gamma_{1ij} \end{array}$$

where β_00*j*_ represents the mean initial status for school *j*, and γ_0*ij*_ is the variation in initial status among students within-school. β_10*j*_ is the mean growth for school *j*, and γ_1*ij*_ is the variation in growth rate among students within-school. γ_0*ij*_ and γ_1*ij*_ are assumed to be in multivariate normal distribution, each with a mean of 0, and some variance (i.e., $\sigma_{r0}^{2}$ and $\sigma_{r1}^{2}$, respectively) and covariance among them (i.e., $\sigma_{r01}^{2}$). The variances of γ_0*ij*_ and γ_1*ij*_ indicate the extent to which students within a school vary from school mean initial status and growth rate. The level-3 equations are as follows

(4) $$\begin{array}{r} \beta_{00j}=\gamma_{000}+u_{00j} \end{array}$$

(5) $$\begin{array}{r} \beta_{10j}=\gamma_{100}+u_{10j} \end{array}$$

where γ_000_ represents the grand mean initial status, and *u*_00*j*_ is the variation in initial status across schools. γ_100_ represents the grand mean growth rate, and *u*_10*j*_ is the variation in growth rate across schools. *u*_00*j*_ and *u*_10*j*_ are assumed to be in multivariate normal distribution, each with a mean of 0, and some variance (i.e., $\sigma_{u00}^{2}$ and $\sigma_{u10}^{2}$, respectively) and covariance among them (i.e., $\sigma_{u01}^{2}$). The variances of *u*_00*j*_ and *u*_10*j*_ indicate the extent to which schools vary with respect to the grand mean initial status and growth rate of the whole sample. To summarize, the three-level growth model described here combines longitudinal and multilevel features because level-1 describes each student's growth in outcomes over time (i.e., the longitudinal feature), level-2 captures the variation across students in growth parameters (i.e., initial status and growth rate) within a schools, and level-3 captures variation in initial status and growth rate among schools (i.e., the multilevel features).

When researchers conduct a three-level growth model analysis, the question of sufficient sample size for adequate statistical power and accurate estimates of parameters arise. If there are relatively small sample sizes, biased estimates may be obtained and the statistical test may lack sufficient power to detect the effect. A review of multilevel studies in education, psychology and sociology demonstrated the difficulties of achieving sufficient sample sizes in applied research (Dedrick et al., 2009). According to this review, of 99 multilevel studies from 13 journals (1999–2003), 21% did not meet the sample size recommendation. Although this result is limited to two-level models, we can infer a similar conclusion from three-level growth models. Thus, this finding suggests that researchers may not be aware of sample size guidelines. Though many researchers acknowledge the importance of sample size, budget constraints and limited time make it difficult to collect enough samples in applied research because receiving approval from several schools can be unfeasible. Therefore, it is important that researchers are aware of the possibility of unreliable results when sample sizes are small.

In this paper, simulation studies are used to examine the effects of different sample sizes at each level on the accurate estimates and adequate power in three-level growth models in which time points are nested within individuals and individuals are nested within groups. Moreover, various ICC values will be considered to reflect realistic conditions. Monte Carlo methods were used to investigate model convergence rate, parameter bias, mean square error (MSE), 95% coverage rate and the statistical power of the tests. By examining three-level growth models, this study can provide sample size guidelines for researchers who are interested in using a longitudinal design.

[Research Question]

1. What are sufficient level-1, level-2, and level-3 sample sizes for accurate parameter and standard error estimates and adequate power when estimating a three-level growth model?
2. How do study conditions affect parameter and standard error estimates and power when estimating a three-level growth model?

## Literature Review

The question of how many individuals per group and how many groups should be sampled to obtain accurate estimates (e.g., fixed effects estimates, random effects estimates, standard errors) from two-level MLM has been addressed in several studies (Kreft, 1996; Maas and Hox, 2004, 2005; Snijders and Bosker, 2012). Depending on the researcher's interest in estimates, required sample sizes maybe changed. Compared to fixed effects, random effect estimates require considerably more groups in order to captures statistically significant variation across groups (Maas and Hox, 2005; Clarke and Wheaton, 2007). For example, studies have demonstrated no bias in the estimates of fixed effects with 30 clusters (Maas and Hox, 2004; Clarke, 2008; Bell et al., 2014). The same results have been observed for the standard errors of the fixed effects that 30 groups are required to obtain for unbiased standard errors (Maas and Hox, 2004, 2005). However, with random effects, overestimated estimates are obtained with small group sizes (Maas and Hox, 2004, 2005; Clarke, 2008). Maas and Hox (2005) demonstrated that with 30 groups, the non-coverage rate for level-2 variance is around 9%. Clarke (2008) examined data sparseness in MLM and illustrated that five observations per group with 200 groups are required to obtain reliable results for random effects. In addition, the results of simulation studies regarding power have been addressed. For the power to detect the effect, Bassiri (1988) noted that 30 groups with 30 individuals per group are required. Scherbaum and Ferreter (2009) also demonstrated that given an effect size of 0.5, the power to detect the effect of a group-level predictor exceeded 0.8 with 30 groups with a size 30. However, Bell et al. (2014) maintained that the commonly cited rule of 30 groups and 30 individuals per group would likely not guarantee a statistical power of 0.8 for the fixed effects at each level of the model. Moreover, in multilevel models, the size of the intraclass correlation (ICC) influences the power to detect covariate effects (Goldstein, 1995). When there is a higher ICC, larger differences between groups exist, and that group variance explains the variance of the group effect, thus reducing the power to detect an effect (Heck and Thomas, 2015).

While several sample size studies have been conducted on the two-level MLM, little is known about sample size requirements for the three-level MLM (McNeish and Stapleton, 2016). De Jong et al. (2010) performed an a priori power analysis for three-level longitudinal models. They used a routine outcome monitoring (ROM) data consisting of 1,966 measurements of patient functioning within 610 patients, who were treated by 109 therapists. The ICCs at the patient and therapist levels were 0.75 and 0.18. In the results, they indicated that increases in sample size at level 2 and 3 improve power, and that increasing the number of measurements did not increase power very much. Li and Konstantopoulos (2016) experimented with methods for power analysis in three-level polynomial change models for cluster randomized designs. They found that power increased as the number of measurement occasions, the number of individuals in each group and the number of groups increased. While all other things being equal, the number of level 3 units influences power more than the number of level 2 units or the number of measurement occasions of the study. These studies performed a power analysis and reported the required sample size amount to achieve a power >0.80. However, in addition to power, it is necessary to have adequate sample sizes at each level to obtain accurate estimates of parameters and standard errors. Even if sample sizes are large enough to obtain adequate power, they may not be sufficient for accurate parameter estimates (Maxwell et al., 2008). In some cases, this results in larger sample sizes than are necessary for enough power. Otherwise, model parameters and standard errors could be seriously biased, thus inflating the Type I error. Moreover, De Jong et al. (2010) used real data where the ICC at level-3 was rather high instead of other naturalistic data. A higher ICC means that there is a larger difference between groups and that the group variance explains part of the variance of the effect, thus reducing the power to detect that effect. When lower ICC values exist, a small sample size is sufficient for power. Thus, various ICC values should be considered in simulation to determine sufficient sample size. In conclusion, to investigate adequate sample sizes for a three-level growth model for accurate estimates and power, this study conducts a simulation under a variety of conditions. By examining a three-level growth model, this study can provide sample size guidelines for researchers who are interested in longitudinal design.

## Methods

In this paper, to generate the model a simple three-level growth model is used, with one explanatory variable at the individual level and one explanatory variable at the group level. The first level for change over time of level 2 unit *i* in group *j* can be expressed as follows:

(6) $$\begin{array}{rcl} \text{level }1:Y_{tij} & = & \pi_{0ij}+\pi_{1ij}time_{tij}+e_{tij} \end{array}$$

(7) $$\begin{array}{r} \text{level }2:\pi_{0ij}=\beta_{00j}+\beta_{01j}X_{ij}+\gamma_{0ij} \end{array}$$

(8) $$\begin{array}{r} \pi_{1ij}=\beta_{10j}+\beta_{11j}X_{ij}+\gamma_{1ij} \end{array}$$

(9) $$\begin{array}{r} \text{level }3:\beta_{00j}=\gamma_{000}+\gamma_{001}Z_{j}+u_{00j} \end{array}$$

(10) $$\begin{array}{r} \beta_{01j}=\gamma_{010}+u_{01j} \end{array}$$

(11) $$\begin{array}{r} \beta_{10j}=\gamma_{100}+\gamma_{101}Z_{j}+u_{10j} \end{array}$$

(12) $$\begin{array}{r} \beta_{11j}=\gamma_{110}+u_{11j} \end{array}$$

(13) $$\begin{array}{l} \text{Combined}:Y_{tij}=\gamma_{000}+\gamma_{001}Z_{j}+u_{00j}\\ \;+\left(\gamma_{010}+u_{01j}\right)X_{ij}+\gamma_{0ij}+(\gamma_{100}+\gamma_{101}Z_{j}\\ \;+u_{10j}\left(\gamma_{110}+u_{11j}\right)X_{ij}+\gamma_{1ij})time_{tij}+e_{tij} \end{array}$$

Three conditions are varied in the simulation: (1) intraclass correlation at group level (ICC: 0.05, 0.15), (2) level-2 sample sizes (group sizes: 10, 30), (3) level-3 sample sizes (number of groups: 30, 50, 100). For the three-level growth model, ICC is calculated for level-2 and level-3. According to Spybrook et al. (2011), repeated measures (level-2) usually have high ICC values that range between 0.5 and 0.7. Otherwise, the ICC values of level-3 range between 0.05 and 0.15, which that correspond to the lowest and overall mean ICC obtained by Hedges and Hedberg (2007) who reported the typical ICC in applied behavioral research. Based on previous research, the first set of ICC was set to 0.45 for level-1, 0.5 for level-2, and 0.05 for level-3. In the second set of ICC, level-1 ICC was set to 0.35, level-2 ICC was kept same, 0.5, and level-3 ICC was set to 0.15. In the three-level growth model, there are different sample sizes at each level: the number of measurements per individual (level-1), the number of individuals per group (level-2), and the number of groups (level-3). To analyze the growth model, at least three repeated measures per individual for linear change are required. If researchers want to estimate a quadratic growth model, at least four time points are required because there are more parameters in the model. Therefore, in this simulation study, the level-1 sample size is set to 4. In the case of the level-2 sample size, 30 observations are standard in educational research. Considering this tendency in the literature, the level-2 sample size is set to 10 for the lowest group size and 30 for highest group size. Similarly, for the level-3 sample size, 30 is the smallest acceptable number according to Kreft and de Leeuw (1998). For an upper limit, 100 groups were chosen according to simulation research by Hox and Maas (2001) who concluded that 100 groups are adequate for unbiased estimates. Therefore, in this simulation, a total 2 × 3 × 2 = 12 conditions will be examined.

For each condition, 500 simulated data sets were generated using Mplus 7.4 (Muthén and Muthén, 1998–2015), assuming normally distributed residuals. Multilevel data were generated by using TYPE = THREELEVEL RANDOM command. The regression coefficients are defined as follows: 0.5 for the intercept, and 0.3 (a medium effect size) (Cohen, 1998) for all regression slopes. The residual variance $\sigma_{r}^{2}$ at the individual level is 2.0. To simplify the model, the covariances between residuals are set to zero. In applied research, it is common for the data to include different numbers of students per school, which is an unbalanced design. Therefore, students were differently assigned to schools (5-15/15-45). Maximum likelihood (ML) is used in the analysis of the generated data.

To compare all conditions of the three-level model, several outcomes will be examined: model convergence rate, relative parameter bias, mean square error (MES), 95% coverage rate and power. The convergence rate is the proportion of the number of properly converged replications to the total number of replications (*n* = 500). The defined model results are considered successfully converged when there is no negative variance or a singular matrix and no errors are reported by Mplus. To evaluate the accuracy of the estimates of the fixed effects and random effects from the estimating models, relative parameter bias can be calculated. The formulas for relative parameter bias are as follows

(14) $$\begin{array}{r} Bias\left(\theta_{i}\right)=\frac{\bar{\theta_{i}}-\theta_{i}}{\theta_{i}} \end{array}$$

where θ_*i*_ is the population value of parameter *i* and $\bar{\theta_{i}}$ is the parameter estimate averaged across the 500 replications in each condition (Hoogland and Boomsma, 1998). Hoogland and Boomsma (1998) concluded that a relative parameter bias up to 5% is tolerable. If parameter estimates under- or overestimate the population value by more than 5%, estimation is at risk for inaccuracy. Because estimation accuracy is also reflected by the standard error, a growing number of scholars have suggested MSE because it combines bias and standard error (Zitzmann et al., 2021). To measure the overall estimation accuracy, MSE, the sum of the squared bias and variance of the parameter estimate, is considered another criterion. Unlike the relative parameter bias, MSE does not have an acceptable range; therefore, a smaller MSE represents that estimates are obtained in a more reliable and accurate way. The coverage probability of a confidence interval is the proportion of replications where the true parameter value is captured in each condition. Coverage indicates how well the parameters and standard errors are estimated. Coverage rates between 91 and 98% are considered acceptable (Muthén and Muthén, 2002). The power can be calculated as the proportion of replications that reject the null hypothesis (α = 0.05). Following common guidelines, a statistical power of 0.80 is appropriate (Cohen, 1992; Muthén and Muthén, 2002). This means that for population parameters different from zero, a significant effect should be detected in at least 80% of the generated samples.

## Results

### Convergence Rate

The overall rate of model convergence varied from 99.8 to 100%, with no negative variance estimates in all converged models. With 30 groups and a group size of 10, the convergence rates were 99.8%. For 50 and 100 groups, all replications were successfully converged. The convergence rate improved with either an increase in the number of groups or an increase in the group size.

### Relative Parameter Bias

**Figure 1:**
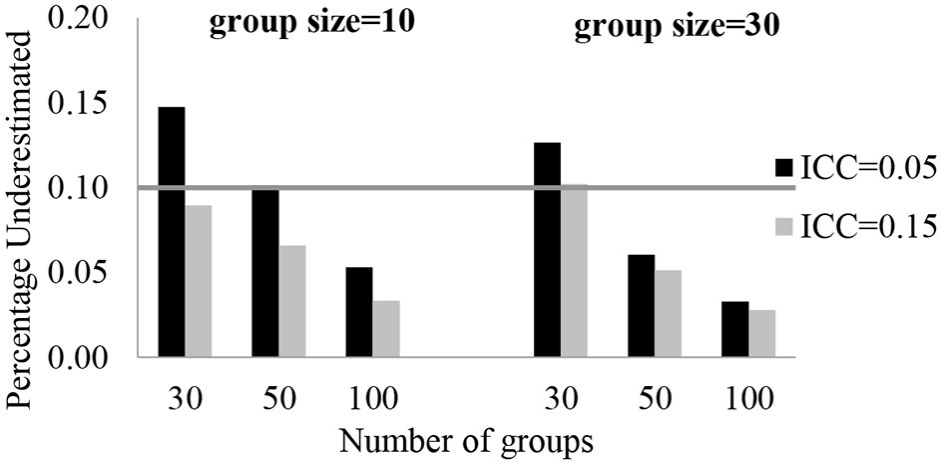
Relative bias for level-3 variance component (*u*_00*j*_).

The number of groups and group size had no effect on the fixed effect estimates, the intercept and regression slopes, under all conditions. The average bias was smaller than 5%. The largest bias of estimates was found with the smallest sample sizes with the highest ICC. The relative bias was 4.4%, and it was a negligible bias. The relative bias value for the random effect estimates were estimated with small bias. Except for a level-3 variance component, random effect estimates were not biased in all conditions. With 30 groups, the relative bias for the 3-level residual variance (*u*_00*j*_) was 10.2–14.8%. The largest relative bias was for *u*_00*j*_ and it was under the condition when ICC is 0.05, the number of groups is 30, and group sizes are 10. In Figure 1, each plot shows a different group size while the number of groups is on the x-axis, and the relative bias of estimates is on the y-axis. The gray line indicates a reference line for the cut-off criteria (0.10 for percent underestimated).

### Mean Square Error

To assess the overall estimation accuracy, the MSE was obtained. The MSEs for the fixed and random effect estimates are presented in Table 1. Overall, the MSEs ranged from 0.001 to 0.055. The largest MSE was found with the smallest sample sizes, such as 30 groups and a group size of 10. The MSE decreased when the number of groups and group sizes increased.

**Table 1:** Mean Square Errors (MSEs) for fixed and random effect estimates.

**Parameter**	**ICC**	**Number of groups**					
		**30**		**50**		**100**	
		**Group sizes**					
		**10**	**30**	**10**	**30**	**10**	**30**
γ_000_	0.05	0.016	0.010	0.011	0.007	0.005	0.003
	0.15	0.029	0.023	0.021	0.015	0.009	0.007
γ_001_ (Z)	0.05	0.018	0.012	0.010	0.006	0.005	0.003
	0.15	0.034	0.027	0.019	0.014	0.009	0.007
γ_010_(X)	0.05	0.017	0.010	0.011	0.006	0.005	0.003
	0.15	0.017	0.010	0.011	0.006	0.005	0.003
γ_100_ (Time)	0.05	0.011	0.008	0.008	0.005	0.003	0.003
	0.15	0.011	0.008	0.007	0.005	0.003	0.003
γ_101_ (TimeZ)	0.05	0.001	0.009	0.007	0.005	0.004	0.003
	0.15	0.013	0.009	0.007	0.005	0.004	0.003
γ_110_ (TimeX)	0.05	0.012	0.009	0.006	0.005	0.004	0.003
	0.15	0.012	0.008	0.006	0.005	0.004	0.003
*e*_*tij*_	0.05	0.011	0.003	0.005	0.002	0.003	0.001
	0.15	0.007	0.002	0.003	0.001	0.002	0.001
γ_0*ij*_	0.05	0.055	0.014	0.034	0.009	0.018	0.004
	0.15	0.051	0.013	0.032	0.008	0.016	0.004
γ_1*ij*_	0.05	0.009	0.003	0.006	0.002	0.003	0.001
	0.15	0.007	0.002	0.005	0.001	0.002	0.001
*u*_00*j*_	0.05	0.015	0.006	0.009	0.004	0.004	0.002
	0.15	0.055	0.033	0.033	0.019	0.015	0.011
*u*_01*j*_	0.05	0.016	0.007	0.009	0.004	0.005	0.002
	0.15	0.016	0.007	0.009	0.003	0.004	0.002
*u*_10*j*_	0.05	0.007	0.004	0.005	0.002	0.002	0.001
	0.15	0.007	0.004	0.005	0.002	0.002	0.001
*u*_11*j*_	0.05	0.008	0.005	0.005	0.002	0.002	0.001
	0.15	0.007	0.004	0.004	0.002	0.002	0.001

### 95% Coverage Rate

**Figure 2:**
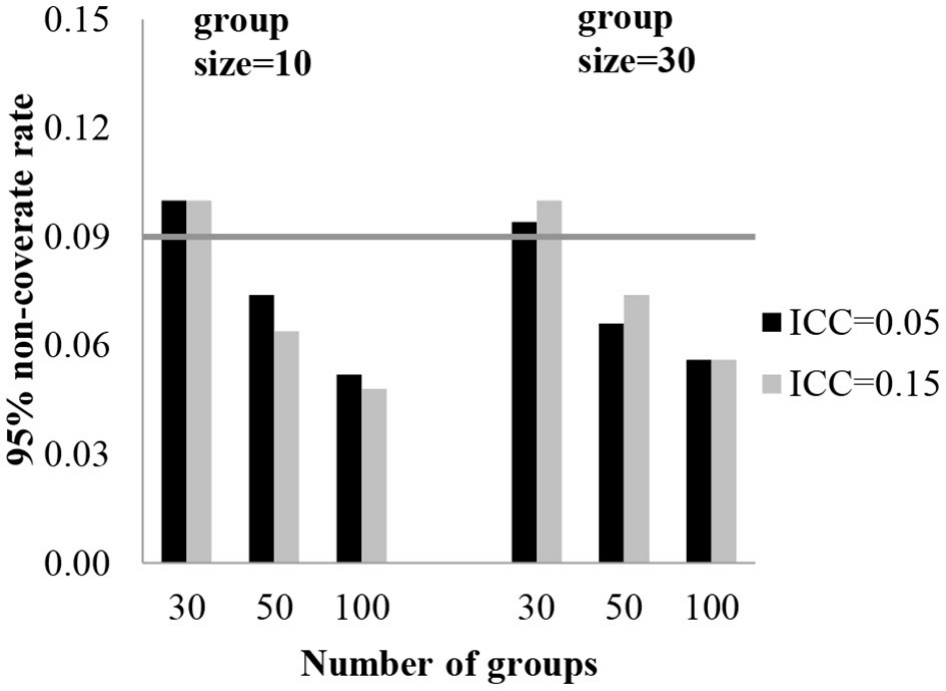
95% coverage rate for level-3 fixed effect standard error (γ_001_).

**Figure 3:**
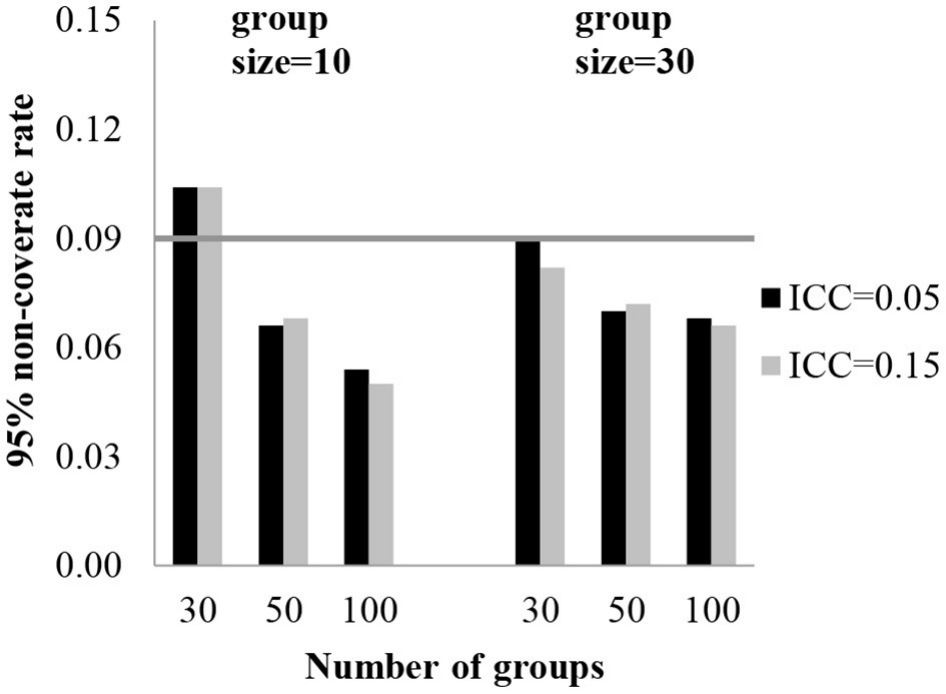
95% coverage rate for level-3 fixed effect standard error (γ_101_).

To assess the accuracy of the standard errors, a 95% coverage rate was obtained. The 95% coverage rates for the fixed effect estimates are presented in Table 2. The standard error estimates for the fixed effect of level-3 were biased with 30 groups. With 30 groups, the coverage rates were 89.6–90.6% for the regression coefficients of level-3 (γ_001_, γ_101_). Table 2 shows the effect of the conditions on the coverage rate for standard errors of the fixed coefficients. In Figures 2, 3, each plot represents γ_001_ and γ_101_, while the number of groups is on the x-axis and 95% coverage rates are on the y-axis. The gray line indicates a reference line for the cut-off criteria (0.91 for 95% coverage rate).

**Table 2:** 95% coverage rate for fixed effect standard errors.

**Parameter**	**ICC**	**Number of groups**					
		**30**		**50**		**100**	
		**Group sizes**					
		**10**	**30**	**10**	**30**	**10**	**30**
γ_000_	0.05	0.934	0.938	0.914	0.936	0.956	0.944
	0.15	0.944	0.934	0.918	0.932	0.948	0.940
γ_001_ (Z)	0.05	0.900	0.906	0.926	0.934	0.948	0.944
	0.15	0.900	0.900	0.936	0.926	0.952	0.944
γ_010_ (X)	0.05	0.944	0.940	0.940	0.940	0.940	0.948
	0.15	0.938	0.944	0.942	0.944	0.940	0.952
γ_100_ (Time)	0.05	0.942	0.944	0.944	0.944	0.948	0.932
	0.15	0.940	0.940	0.944	0.942	0.946	0.932
γ_101_ (TimeZ)	0.05	0.896	0.910	0.934	0.930	0.946	0.932
	0.15	0.896	0.918	0.932	0.928	0.950	0.934
γ_110_ (TimeX)	0.05	0.934	0.920	0.954	0.942	0.940	0.944
	0.15	0.940	0.916	0.954	0.946	0.940	0.942

*The shaded cells indicate meaningfully biased values*.

The effect of the number of groups and group sizes on the standard errors of the variance components is sufficiently large. The 95% coverage rates for random effect standard errors are presented in Table 3. With 30 groups, the coverage rates for the level-3 variances (*u*_00*j*_, *u*_01*j*_, *u*_10*j*_ *and u*_11*j*_) were 79.8–88.2%. With 50 groups, the coverage rates for the level-3 variances were 85.6–91.0%. With 100 groups, the coverage rates for the level-3 variances were 89.8–92.8%.

**Table 3:** 95% coverage rate for random effect standard errors.

**Parameter**	**ICC**	**Number of groups**					
		**30**		**50**		**100**	
		**Group sizes**					
		**10**	**30**	**10**	**30**	**10**	**30**
*e*_*tij*_	0.05	0.922	0.938	0.962	0.938	0.946	0.946
	0.15	0.926	0.934	0.962	0.934	0.952	0.946
γ_0*ij*_	0.05	0.928	0.940	0.934	0.940	0.946	0.944
	0.15	0.924	0.944	0.930	0.942	0.946	0.938
γ_1*ij*_	0.05	0.940	0.940	0.914	0.944	0.944	0.956
	0.15	0.932	0.942	0.924	0.938	0.946	0.950
*u*_00*j*_	0.05	0.848	0.798	0.868	0.882	0.910	0.902
	0.15	0.852	0.808	0.862	0.874	0.914	0.898
*u*_01*j*_	0.05	0.882	0.848	0.874	0.904	0.908	0.928
	0.15	0.868	0.850	0.876	0.902	0.922	0.918
*u*_10*j*_	0.05	0.850	0.822	0.856	0.908	0.912	0.916
	0.15	0.858	0.824	0.862	0.910	0.906	0.920
*u*_11*j*_	0.05	0.858	0.826	0.876	0.902	0.916	0.906
	0.15	0.856	0.832	0.876	0.904	0.916	0.902

*The shaded cells indicate meaningfully biased values*.

### Power

**Figure 4:**
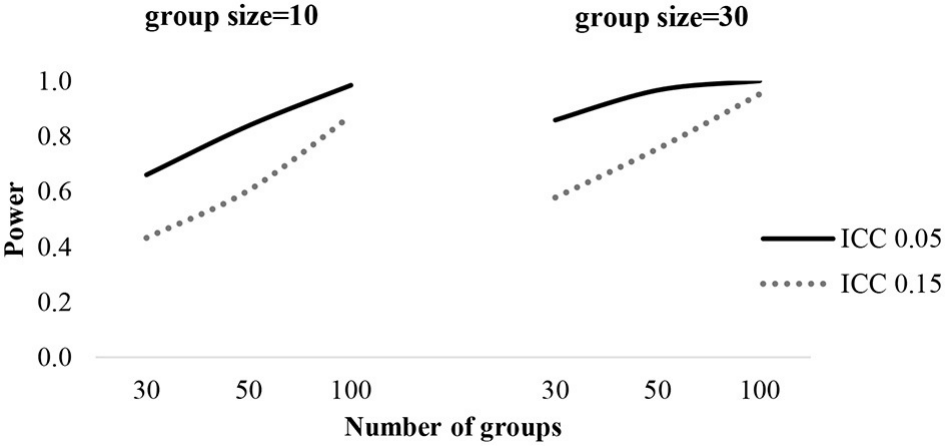
Statistical power of Z coefficients (γ_001_).

For the statistical power test of each fixed effect, the general pattern of results is presented in Table 4. Across all effects, power rates exceeded 0.8 with a group size of 50, ranging from 0.874 to 1.000, except level-3 predictors (Z). Power estimates for the fixed effect Z reached the 0.8 level with groups of a sample size of 100. When ICC is 0.05, the power of Z would be 0.86 with 30 groups containing 30 individuals per group, while power exceeds 0.8 with 100 groups of a sample size of 10 with a 0.15 ICC. The power of level-2 predictors (X) exceeds 0.8 with 50 groups containing 30 individuals in each group. In order to obtain sufficient power, more than 50 groups and 30 individuals per group are required. Figure 4 shows the power curve of level-3 predictors (Z) with the number of groups, group sizes and ICC.

**Table 4:** Power for fixed effect.

**Parameter**	**ICC**	**Number of groups**					
		**30**		**50**		**100**	
		**Group sizes**					
		**10**	**30**	**10**	**30**	**10**	**30**
γ_000_	0.05	0.978	1.000	1.000	0.998	1.000	1.000
	0.15	0.832	0.916	0.954	0.976	0.998	1.000
γ_001_ (Z)	0.05	0.660	0.858	0.838	0.966	0.984	1.000
	0.15	0.432	0.578	0.604	0.754	0.874	0.952
γ_010_ (X)	0.05	0.508	0.672	0.710	0.886	0.940	0.992
	0.15	0.508	0.680	0.698	0.894	0.942	0.992
γ_100_ (Time)	0.05	0.652	0.786	0.860	0.938	0.988	1.000
	0.15	0.666	0.792	0.870	0.944	0.990	1.000
γ_101_ (TimeZ)	0.05	0.790	0.890	0.936	0.992	1.000	1.000
	0.15	0.802	0.898	0.940	0.992	1.000	1.000
γ_110_ (TimeX)	0.05	0.644	0.816	0.840	0.950	0.982	0.998
	0.15	0.658	0.826	0.870	0.948	0.982	0.998

*The shaded cells indicate power < 0.8*.

## Discussion

When educational researchers are interested in the changes in the academic performance of students who are nested in schools, a three-level growth model is required for analysis. Recently, application of a three-level model has grown in education, psychology, and social science research. However, to achieve reliable results in multilevel models, it is important to obtain adequate sample sizes. If there are relatively small sample sizes, estimated parameters might be biased, and the statistical power to detect an effect may be insufficient. Simulation studies in various conditions have been conducted to provide sample size guidelines and to indicate the possibility of unreliable results when sample sizes are small. The purpose of this paper was to perform simulation studies for three-level growth models and to illustrate the effect of the ICC, level-2 and level-3 sample size on the accuracy of estimates and power. This study performed a Monte Carlo simulation with 12 conditions: (1) level-2 sample size (10, 30), (2) level-3 sample size (30, 50, 100) (3) ICC at level-3 (0.05, 0.15).

In this study, two research questions were suggested to find adequate sample sizes in the three-level growth model. First, what are sufficient level-1, level-2, and level-3 sample sizes for accurate parameter and standard error estimates and adequate power when estimating a three-level growth model? Second, how do the study conditions affect parameter and standard error estimates and power when estimating a three-level growth model?

Results indicated that the fixed effects, the intercept and regression slopes, are all estimated without bias under all simulated conditions. The sample size combination of 4 time points among 10 individuals within 30 groups was sufficient to estimate an unbiased fixed effect. This finding aligns with the results of previous 2-level model studies that demonstrated no bias in the estimates of the fixed effects with 30 groups (Maas and Hox, 2004; Clarke, 2008; Bell et al., 2014). However, the standard error estimates for the fixed effect of level-3 were slightly biased with 30 groups. With 30 groups, the coverage rates were 89.6–90.6% for the regression coefficients of level-3 (γ_001_, γ_101_).

The estimates of random effects, except for level-3 variance, were not biased under all conditions. With 30 groups, the relative bias for the 3-level residual variance (*u*_00*j*_) was 10.2–14.8%. According to the results, to obtain accurate estimates for random effects, more than 30 groups with 30 individuals per group are needed. Compared to the common rule of 30/30 proposed by previous research for a two-level model, this result demonstrates that slightly more samples are required for a three-level model (Kreft, 1996; Maas and Hox, 2004, 2005). Otherwise, a 95% coverage rate for random effects is underestimated within level-3 variances. As previously researched, standard error estimates of group-level variance are the most influenced model estimate when the sample size is small (Maas and Hox, 2005; Clarke, 2008). The coverage rates for level-3 standard error estimates are biased even in the 100 groups, indicating that at least 100 groups are needed to obtain reliable confidence (McNeish and Stapleton, 2016).

For adequate power, more than 50 groups and 30 individuals per group are required when coefficients have medium effect size (*d* = 0.3). As previous studies illustrated, level-3 sample sizes create greater effects than level-2 sample sizes in increasing statistical power (De Jong et al., 2010; Li and Konstantopoulos, 2016). This indicates that researchers are suggested to collect more schools rather than students. Moreover, the higher ICCs are, the larger sample sizes are required to obtain sufficient power for group level effects.

In conclusion, the results from this study provide researchers with helpful guidelines regarding the effect of certain conditions on their results. The sample size recommendations from this study indicate that the three-level growth model can be used with small sample sizes, depending on the parameter of interest. When researchers are interested in estimating a fixed effect, the standard error estimates for the fixed effect of level-3 could be slightly underestimated with 30 groups, thus inflating the Type I error. Therefore, at least 50 groups are required for accurate fixed effect estimates. Compared to fixed effects, random effect estimates require at least 100 groups in order to capture statistically significant variations across groups.

Compared to previous studies, it appears that researchers can gain information about sample size for three-level growth model with unbalanced designs and varied ICC. Moreover, this study examined both the parameter estimates and power to obtain reliable results not only for individual research but also for building a cumulative science (Maxwell et al., 2008).

However, as with any other simulation studies, this study has some limitations. One major limitation is that this research used one predictor at each level. In applied research, usually more than one predictor is analyzed. If more variables are added, the model becomes complex and more parameters should be estimated. In further research, it is advisable to examine more complex multilevel models.

Second, this study used maximum likelihood (ML) for estimating the multilevel model. However, it has been argued and shown that Bayesian estimation is less demanding and can work well even in very small samples (Hamaker and Klugkist, 2011; Hox et al., 2012). Thus, in future research, Bayesian estimation can be considered for reducing the sample size requirements in three-level growth models.

Third, the outcome variable used in this study was a continuous variable and assumed normal distribution. However, binary, ordinal and nominal outcomes requiring multilevel logistic regression or poisson regression have not been addressed. In previous research on two-level models, simulations have been conducted for binary and mixed outcomes (Moineddin et al., 2007; Austin, 2010; Bell et al., 2014). Thus, other types of outcome variables could be explored to provide more detailed guidelines to researchers.

Despite these limitations, the results of this study extend the understanding of adequate sample sizes for a three-level growth model given various conditions. This study indicates that required sample sizes can be different, according to researchers' interest in parameter estimates (i.e., fixed effect, random effect) at each level, ICC and statistical power. In this regard, researchers are encouraged to consider sample sizes based on their research questions, especially when there are budget and time limitations.

## Data Availability Statement

The raw data supporting the conclusions of this article will be made available by the authors, without undue reservation.

## Author Contributions

EL designed the study, performed the simulations, and wrote the manuscript. SH supervised the directions of this research and contributed to the interpretation of the results. Both authors contributed to manuscript revision, read, and approved the submitted version.

## Conflict of Interest

The authors declare that the research was conducted in the absence of any commercial or financial relationships that could be construed as a potential conflict of interest.
