# Supplementary material for: Adequate Sample Sizes for a Three-Level Growth Model
Source: Front Psychol. 2021 Jul 1;12:685496. doi: 10.3389/fpsyg.2021.685496 (PMC8282204; doi:10.3389/fpsyg.2021.685496)
Supplement: Supplementary file 1 [file Data_Sheet_1.PDF]

Supplementary Material

1 Supplementary Data

Mplus simulation code for Three-Level Growth Model
(Group sizes: 10, Number of groups: 30, ICC: 0.05)

```
TITLE:  This is an example of 3-level regression

MONTECARLO:
        names are y x w z;
        nobservations = 1200;
        nreps = 500;
        SEED = 58453;
        CSIZES = 15[5(4)] 15[15(4)];
        ncsize = 2[1];
        within = x;
        between =(level2) w (level3) z;
        REPSAVE=all;
        SAVE = G30_GS10_ICC0.05_V*.dat;

ANALYSIS:  TYPE = threelevel random;
MODEL POPULATION:
        %within%
        x@1;
        s1 | y on x;
        y*1.8;

        %between level2%
        w@1;
        s2| y on w*.3;
        y*2;
        s12| s1 on w*.3;
        s1*.4;


        %between level3%
        z@1;
        y on z*.3;
        y*.2;
        [y*.5];
        s1 on z*.3;
        s1*.2;
        [s1*.25];
        s2*.2;
```

```
        [s2*.25];
        s12*.2;
        [s12*.25];


MODEL:
        %within%
        s1 | y on x;
        y*1.8;

        %between level2%
        s2| y on w*.3;
        y*2;
        s12| s1 on w*.3;
        s1*.4;

        %between level3%
        y on z*.3;
        y*.2;
        [y*.5];
        s1 on z*.3;
        s1*.2;
        [s1*.25];
        s2*.2;
        [s2*.25];
        s12*.2;
        [s12*.25];

OUTPUT: TECH9;
```

2 Supplementary Figures and Tables

2.1 Supplementary Figures

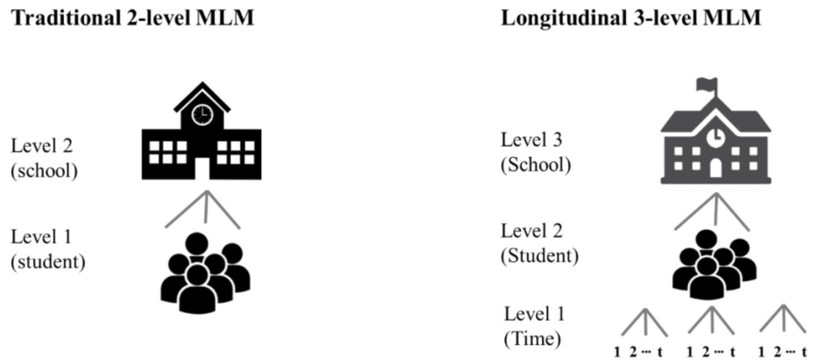

Supplementary Figure 1. Traditional 2-level and longitudinal 3-level multilevel model

2.2 Supplementary Tables

Supplementary Table 1. Relative parameter bias for fixed effect estimates

Parameter	ICC	Number of Groups (level-3)					
		30		50		100	
		Group sizes (level-2)					
		10	30	10	30	10	30
γ_{000}	0.05	-.008	-.011	-.009	.001	.008	.004
	0.15	-.014	-.015	-.012	-.002	.009	.005
γ_{001} (Z)	0.05	.026	-.019	.015	-.024	.009	-.010
	0.15	.044	-.041	.022	-.033	.012	-.012
γ_{010}(X)	0.05	-.018	.036	.004	.005	-.007	-.005
	0.15	-.020	.036	.006	.005	-.007	-.005
γ_{100} (Time)	0.05	-.012	-.012	-.017	-.014	.000	-.002
	0.15	-.012	-.013	-.016	-.014	.000	-.002
γ_{101} (TimeZ)	0.05	.000	.011	.010	.005	-.006	.015
	0.15	.000	.010	.009	.005	-.005	.015
γ_{110} (TimeX)	0.05	-.002	-.014	-.013	-.020	-.020	.000
	0.15	-.003	-.014	-.014	-.020	-.018	.000

Supplementary Table 2. Relative parameter bias for random effect estimates

Parameter	ICC	Number of Groups					
		30		50		100	
		Group sizes					
		10	30	10	30	10	30
e_{tij}	0.05	.001	-.001	.002	-.001	-.001	-.001
	0.15	.001	-.001	.002	-.001	-.001	-.001
γ_{0ij}	0.05	.001	.002	-.003	.000	-.005	-.001
	0.15	.000	.002	-.004	.000	-.005	-.001
γ_{1ij}	0.05	.011	.005	.008	.001	.008	.000
	0.15	.010	.004	.007	.000	.006	.000
u_{00j}	0.05	.148	.127	.102	.061	.053	.033
	0.15	.090	.102	.066	.051	.033	.028
u_{01j}	0.05	.060	.031	.113	.041	.029	.017
	0.15	.066	.032	.114	.041	.030	.017
u_{10j}	0.05	.092	.090	.068	.040	.050	.026
	0.15	.089	.088	.065	.039	.050	.025
u_{11j}	0.05	.059	.074	.046	.032	.022	.016
	0.15	.057	.072	.044	.031	.021	.016
